# Adherence to highly active antiretroviral therapy and associated factors among children at the University of Gondar Hospital and Gondar Poly Clinic, Northwest Ethiopia: a cross-sectional institutional based study

**DOI:** 10.1186/1471-2458-14-875

**Published:** 2014-08-26

**Authors:** Berihun Assefa Dachew, Tadis Brhane Tesfahunegn, Anteneh Messele Birhanu

**Affiliations:** Department of Epidemiology and Bio-statistics, University of Gondar, College of medicine and Health science, Institute of public health, P.O. Box: 196, Gondar, Ethiopia; Department of Nursing, Axum University, P.O. Box: 1010, Axum, Ethiopia; Department of Nursing, University of Gondar, College of Medicine and Health Science, P.O. Box: 196, Gondar, Ethiopia

**Keywords:** Adherence, Antiretroviral therapy, Children

## Abstract

**Background:**

The efficacy of antiretroviral therapy (ART) in suppressing viral replication and delaying the progress of the acquired immunodeficiency syndrome (AIDS) is related to optimal adherence. Adherence is a challenge in all HIV infected people on ART. It is especially a concern in children because of factors relating to children such as age, disclosure status of HIV sero status, and understanding of the medication. This study assessed the level of adherence to highly active antiretroviral therapy and its associated factors among children in Gondar University Hospital and Gondar Poly Clinic, Northwest Ethiopia.

**Methods:**

Institutionally based cross-sectional study design was conducted from January-to March 2012. Simple random sampling technique was used to select study participants and a total of 342 study subjects were included in the study. Bivariate and multivariate logistic regressions were performed to identify associated factors with adherence to highly active antiretroviral therapy. Odds ratios with 95% confidence interval were computed to determine the level of significance.

**Results:**

The overall ART adherence among children was found to be 90.4%. Age of the child [AOR = 0.37 (95% CI: 0.31, 0.46)], disclosure of the child’s HIV status to the child [AOR = 0.27 (95% CI: 0.24, 0.32)], and knowledge of caregivers about ART medication [AOR = 4.7 (95% CI (3.7, 5.6)], were independently associated with adherence.

**Conclusion:**

Adherence rate to ART was found to be high. Disclosure of the child’s HIV sero status to the child, the age of the child and the knowledge of the caregivers towards ART were factors associated with adherence.

## Background

Acquired immunodeficiency syndrome (AIDS) is a major threat to the world’s population and it is the most devastating disease humankind has ever seen. In 2010, there were an estimated 33.3 million people living with HIV/AIDS in the world, of which more than 90% live in developing countries. Among these, children less than 15 years of age account for around 2.5 million. Africa alone contains two-thirds of the world’s total prevalence [1]. Sub-Saharan Africa is more heavily affected by HIV/AIDS than any other region of the world [2]. Of all deaths due to AIDS in the world, 1.6 million occurred in Sub-Saharan Africa [3]. In Ethiopia, there were about 1.2 million people living with HIV in the year 2010 alone and the overall prevalence is 2.3% [4]. A total of 258,264 people living with HIV/AIDS are in need of ART, and 6.1% are children [5]. Free antiretroviral treatment (ART) service was launched in Ethiopia in January 2005 and hospitals began providing free antiretroviral drugs in March 2005. The government focused on accelerated access to ART in June 2006. This accelerated access, especially in health centers, was not accompanied by an equally rapid rise in ART uptake as expected [6]. Although resources are expanding in the overall HIV situation, preventing and treating pediatric HIV infection in resource-limited settings remains extremely challenging [7].

Despite the fact that the treatments are not a cure and present new challenges with respect to side-effects and drug resistance, these treatments have dramatically reduced rates of mortality and morbidity, improved the quality of life of people with HIV/AIDS, and given encouragement to the communities [8]. However, the efficacy of antiretroviral therapy in suppressing viral replication and delaying the progress of AIDS is related to optimal adherence.

Inadequate adherence increases the risk of drug resistance and treatment failure. Therefore, optimum adherence is highly essential for sustainable success to highly active antiretroviral treatment (HAART) [9]. Taking greater than 95% of prescribed doses is recommended for optimal virologic suppression and to minimize the rate of treatment failure [10, 11]. Virologic failure rate of greater than 50% is associated with less than 95% adherence rate [11].

Though adherence is a challenge in all HIV infected people who are on ART, it is a special concern in children. The lack of pediatric formulations, poor palatability, high pill burden or liquid volume, frequent dosing requirements, dietary restrictions and side-effects may hinder the regular intake of required medications [10]. In addition, poor understanding of the need to take the medication by the parents, and parents who may not wish to disclose the HIV status to the child or to others involved in their child’s care are some of the challenges of adherence to HAART in resource limited settings like Ethiopia [10]. Furthermore, the successful treatment of a child requires the commitment and involvement of a responsible caregiver. This may be particularly complicated if the family unit is disrupted as a consequence of adverse health or economic conditions [12] though the burden of numerous pills is expected to be decreased after the initiation of fixed dose combination [12]. Therefore, evaluating the HAART adherence among children living with HIV can provide significant information for policy makers and organizations working in this area to develop appropriate interventions for ART adherence among children. This will achieve better health outcomes and prevent the emergence of HIV drug resistance strains. This study was aimed at assessing the level of adherence and associated factors to HAART in order to improve sustainable outcomes of HAART among children.

## Methods

### Study design

Institutionally based, cross-sectional quantitative study design was conducted at Gondar University Hospital and Gondar Poly Clinic Pediatrics ART Unit.

### Study area and period

The study was conducted from January to March 2012 in Gondar University Hospital and Gondar poly Clinic pediatrics ART Unit.

### Source and study population

All children who were taking HAART and on ART follow up with Gondar University Hospital and Gondar Poly Clinic.

### Inclusion criteria

Children aged 2 months to 15 years receiving HAART for at least 2 months were included in the study. Approximately 742 children fulfilled the inclusion criteria.

### Sampling procedure

Sample size was calculated by using a single population proportion formula with the assumption of 95% level of confidence, 5% marginal error, taking the proportion of adherence rate 80.9% [13] considering 10% non response rate. Using this assumption, out of 742 children on ART, a total 342 children were included in the study using simple random sampling technique (via computer generated random number).

### Data collection tools and procedures

Data was collected by using a pretested and structured questionnaire administered by face to face interviews with caregivers by three trained health care providers. The questionnaire was adapted from pediatric AIDS clinical trial group (PACTG) adherence follow up questionnaire. Adherence was measured utilizing the caregivers’ report following one month of treatment and the number of times they recalled missing doses. Patients/caregivers who reported an intake of more than 95% of the prescribed medications were considered to be adherent. Medical charts were reviewed to determine clinical marker of the children.

### Data quality assurance

Data was collected by using a pre-tested questionnaire by trained health care providers. There was continuous supervision to control the data collection procedure. All the data, from each ART site, was checked for completeness, clarity and consistency by the principal investigators and supervisors during the interview day. Data was intensively cleaned before analysis. The reliability of the tool was checked using Cronbach’s alpha reliability test with a score of 0.78 (95% CI 0.76 -0.801).

### Data processing and analysis

Data was coded and entered in to EPI info version 3.5.3 statistical software and then exported to SPSS Windows version 20 for further analysis. Adherence to HAART was assessed by using self reporting methods.

Bivariate logistic regression was used to check variables associated with the dependent variable. Those variables found to have p-values of ≤0.2 were fitted to multivariate logistic regression to control the effects of confounders. Odds ratios with 95% CI were computed and variables having p-values ≤0.05 in the multiple logistic regression models were considered significantly associated with the dependent variable. Model fitness was checked with the assumptions of Hosmer and Lemeshow goodness of a fit test (P = 0.79).

### Ethical considerations

Ethical clearance was obtained from the Ethical Review Committee of the University of Gondar. Concerned officials at different levels were contacted and permission for the study was secured. Each caregiver/subject was adequately informed about the purpose of the study and written informed consent was obtained from the parents/guardians. Interviews were conducted in private rooms.

## Results

### Socio-demographic characteristics of the caregivers and children

A total of 314 child caregivers responded to the structured questionnaire, a response rate of 91.8%. Of the respondents, 230 (73. 2%) were females. The mean and the median age of the primary caregivers were 37.77 years ± 6.05 and 35, respectively. Three hundred and one (59.59%), were Amhara in ethnicity, 269 (85.7%) were Orthodox in religion. One hundred and four (33.1%) were not able to read and/or write. Two hundred and thirty four (74.5%) of the caregivers had a family size of less than or equal to five. Most of the care givers interviewed 210 (66.9%) were the biological parents of the children. Of the 314 children, 176 (56.1%) were males and 54.7% were age 10–15 years (Table 1).

**Table 1:** **Socio-demographic characteristics of caregivers and children on HAART at Gondar University Hospital and Ploy Clinic April 2012 (n = 314)**

I) Socio-demographic characteristics of caregivers			
Socio-demographic variables		Number (n = 314)	Percent (%)
**Sex**	Male	84	26.8
	Female	230	73.2
**Age**	< 20	5	1.6
	20-39	210	66.9
	40-59	67	21.3
	>60	32	10.2
**Ethnicity**	Amhara	301	95.9
	Tigray	10	3.2
	Oromo	3	1.0
**Marital status**	Married	128	40.8
	Single	66	21
	Widowed	66	21
	Divorced	53	16.9
	Separated	1	0.3
**Educational status**	Illiterate	104	33.1
	Elementary	146	46.5
	Grade 12 +	64	20.4
**Occupational status**	Non employed	95	30.25
	Merchant	65	20.7
	Daily labor	50	15.9
	Farmer	38	12.1
	Civil servant	62	19.7
**Monthly income**	≤ 525 ETB*	189	60.2
	> 525 ETB*	125	39.8
**Family size**	≤ 5	234	74.5
	> 5	80	25.5
**II) Socio-demographic characteristics of children**			
**Socio-demographic variables**		**Number (n = 314)**	**Percent (%)**
**Type of parent**	Biological	210	66.9
	Relative	94	29.9
	Adoptive	10	3.2
**Sex of children**	Male	176	56.1
	Female	138	43.9
**Age of children (in years)**	0-4	28	8.9
	5-9	114	36.3
	10-15	172	54.7
**Educational status of children**	Attending formal education	206	65.6
	Not attending formal education	108	34.4

*Ethiopian Birr.

### Clinical marker of the children on ART

More than half (52.2%) of the children were WHO stage II classification. Seventy one (22.6%) of the children had CD4 count of <200 cells/mm3 at the start of the treatment (Table 2).

**Table 2:** **Clinical markers of HIV infected children on HAART at Gondar University Hospital and Gondar Poly Clinic, North West Ethiopia April 2012 (n = 314)**

Variables	Number (n = 314)	Percent (%)
**WHO Clinical stage**		
Stage I	56	17.8
Stage II	164	52.2
Stage III	92	29.2
Stage IV	2	0.6
**CD4 counts at start of treatment**		
<200	71	22.6
200-499	164	52.2
≥500	79	25.2
**Current CD4 count**		
<200	52	16.5
200-499	138	43.9
≥500	124	39.5

### Knowledge of caregivers towards ARV Medications

Of the respondents, 200 (63.7%), were aware of ART before their child started treatment. For the knowledge assessment questions, 305 (97.1%) of the respondents knew the types of medication their child was taking and 307 (97.8%) of them knew that children taking ART would be required to take the medications for the rest of their life to delay AIDS progression.

The summary knowledge questions revealed that 282 (89.2%) of the respondents had significant knowledge about ART.

### Patient provider relationship

Almost all of the caregivers, 306 (97.5%), felt they have a good relationship with their health care providers and 303 (96.5%) also have open communication. Almost all individuals, 310 (98.7%), reported how helpful the health care providers in the ART unit were in dealing with their medical problems.

### Health care system and clinical setting

All the respondents had access to pharmacies whenever they wanted and were satisfied with the ease of scheduling appointments and confidentiality. Almost all of the respondents, 308 (98.1%), were satisfied by the changes/improvement their child experienced from the treatment.

### Recommended ARV regimen for children

All children were taking fixed dose combination (FDC) pills. More than half (52%) had been taking AZTT/3TC/NVP base regimen and the remaining 28%, 12% and 8% were under AZT/3TC/EFV, d4T/3TC/NVP and d4T /3TC/EFV regimens respectively.

### Adherence assessment

Of the total children taking ART, 182 (57.9%) of them had a history of missing at least one dose in the last month prior to the survey. Adherence to HAART in HIV positive children during the past three and seven days was assessed and 310 (98.7%) and 304 (96.8%) of the children took greater than 95% of the total prescribed doses respectively. According to the care givers report, the level of adherence to HAART among children was 284 (90.4%) in the one month recall period before the survey. Among the caregivers, 182 (57.9%) of them disclosed their child’s sero status to the child.

### Reasons for missing ARV drugs

The common reasons, mentioned by the respondents, for missed doses were forgetfulness 44 (52.3%), medication fatigue 22 (26.2%), caregiver fear of giving the medication witnessed by other people 12 (14.3.3%), caregiver illness 10 (11.9%) and others 6 (7.14%) away from home, transfer related cases, and religious beliefs leading to use of e.g. holy water.

### Factors associated with adherence to HAART

Through multivariate logistic regression; the age of the child [AOR = 0.37(95% CI: 0.31, 0.46)], disclosure of the child’s HIV status to the child [AOR = 0.27(95% CI: 0.24, 0.32)], and caregiver knowledge of ARV medication [AOR = 4.7 (95% CI (3.7, 5.6)], were independently associated with adherence to ART (Table 3).

**Table 3:** **Factors determining adherence among HIV positive children on at Gondar University Hospital and Ploy Clinic April 2012**

Variables	Adhered no (%)	Non adhered no (%)	Crude OR (95% CI)	Adjusted OR (95% CI)	P- value
**Child age**					
0-4	26 (8.3%)	2 (0.64%)	1	1	
5-9	108 (34.4%)	6 (1.9%)	0.52 (0.46, 0.67)	0.42 (0.36, 0.54)	0.01
10-15	150 (47.8%)	22 (7%)	0.52 (0.42, 0.64)	0.37 (0.31, 0.46)	0. 003
**Educational status of care giver**					
Illiterate	92 (29.3%)	12 (3.8%)	1	------------------	
Elementary	132 (42.0%)	14 (4.5%)	1.2 (1.05, 1.65)		
Secondary and above	60 (19.1%)	4 (1.27%)	0.2 (0.15, 0.29)		
**Disclosure**					
Yes	158 (50.3%)	24 (7.6%)	0.31 (0.25,0.37)	0.27 (0.24, 0.32)	0.001
No	126 (40.1%)	6 (1.9%)	1		
**Occupational status of care givers**					
Non employed	87 (27.7%)	8 (2.5%)	1	------------------------	
Employed	197(62.7%)	22 (4.7%)	0.82 (0.61, 0.98)		
**Knowledge of care giver**					
Good	262 (83.4%)	20 (6.4%)	5.95 (4.2, 6.7)	4.7 (3.7, 5.6)	0.001
Poor	22 (7.0%)	10 (3.2%)	1		

[Odd ratios (OR) with 95% Confidence intervals (CI)].

## Discussion

Today HIV-infected infants and children survive to adolescence and adulthood, but the issue of adherence is still a great challenge.

The overall HAART adherence among children, who were followed up in Gondar University Hospital and Gondar Poly Clinic, was 90.4% by self report assessment. The treatment adherence level found in this study was higher in comparison to studies conducted previously in Ethiopia (80.9%) [13], Nigeria (80%) [14] and Togo (80%) [15].

This finding was also higher as compared to studies conducted in Italy and Uganda which revealed the level of adherence ranges from 79%-90% [16–18]. This result can be explained because the children were on fully subsidized ART and also had a good patient/provider relationship (97.5%) with open communication (96.5%). The other explanation is that most of the children didn’t experience any adverse effects of the medication since side effects are known to increase non adherence [19]. However, our findings are almost consistent with a study conducted in New Delhi (91.4%) [20].

In this study, treatment adherence to ART was significantly associated with the age of the children (P < 0.01). As the age of the child increased the level of adherence to treatment decreased by 63%, [AOR = 0.37 (95% CI: 0.31, 0.46)] which is inconsistent with other studies conducted in Nigeria, Togo, Italy and Brazil [8, 14, 15, 21]. This decrease can be explained by care givers delegating treatment responsibilities to the child irrespective of the child’s knowledge of the medications and their importance.

Disclosure is a critical step and has implications for adherence. Starting with disclosure as early as 8–9 years of age and combining it with specific support is important to increase children’s adherence as they get older [16]. However, in our study, the level of adherence was significantly lower [AOR = 0.27 (95% CI: 0.24, 0.32)] in children who were aware of their sero status than in those who unaware of their status (P < 0.01). This finding is in line with other studies [16, 21] but inconsistent with studies from Uganda [22] and Democratic Republic of Congo [23]. These studies suggest correlation with professional and ongoing counseling after disease disclosure to children.

The study also showed that caregivers of children with good knowledge about the disease were 4.7 times [AOR = 4.7 (95% CI (3.7, 5.6)] more likely to adhere to HAART than their counterparts. This finding is similar to another study conducted in another region of Ethiopia [13, 24].

Though our study does not reveal the presence of an association between treatment adherence and type of caregivers, other authors have suggested that children who live in institutions adhere better to treatment than children who live with their biological parents [16]. Foster parents are trained to provide proper care for their adopted children and showed optimal adherence [25]. Health care providers should strengthen adherence counseling during follow up and address proper usage of medication reminders.

### Limitation of the study

This study has some important limitations that should be borne in mind when interpreting the results. Firstly, since the level of adherence was assessed using caregivers self reports, this may have resulted in an overestimation of the adherence level and may also have been prone to recall bias. In addition the data was gathered by interview; therefore there is some potential to social desirability bias. Furthermore, this study did not assess the knowledge of HIV and HAART among the older children and the effect of this knowledge on their adherence. Finally, this study was not triangulated with qualitative methods.

## Conclusion

In this study, the level of adherence to antiretroviral therapy was found to be high. Disclosure of the child’s sero status to the child, age of the child and caregivers knowledge of ARV treatment were factors associated with HAART adherence.

## Authors’ information

Tadis Brhane Tesfahunegn and Anteneh Messele Birhanu are co-authors.
